# Improving Both the Thermostability and Catalytic Efficiency of Phospholipase D from *Moritella* sp. JT01 through Disulfide Bond Engineering Strategy

**DOI:** 10.3390/ijms231911319

**Published:** 2022-09-26

**Authors:** Lilang Li, Xuejing Mao, Fuli Deng, Yonghua Wang, Fanghua Wang

**Affiliations:** School of Food Science and Engineering, South China University of Technology, Guangzhou 510640, China

**Keywords:** phospholipase D, molecular dynamics simulations, disulfide bonds, thermostability, protein engineering, phosphatidic acid

## Abstract

Mining of Phospholipase D (PLD) with high activity and stability has attracted strong interest for investigation. A novel PLD from marine *Moritella* sp. JT01 (MsPLD) was biochemically and structurally characterized in our previous study; however, the short half-life time (*t_1/2_*) under its optimum reaction temperature seriously hampered its further applications. Herein, the disulfide bond engineering strategy was applied to improve its thermostability. Compared with wild-type MsPLD, mutant S148C-T206C/D225C-A328C with the addition of two disulfide bonds exhibited a 3.1-fold *t_1/2_* at 35 °C and a 5.7 °C increase in melting temperature (*T_m_*). Unexpectedly, its specific activity and catalytic efficiency (*k_cat_*/*K_m_*) also increased by 22.7% and 36.5%, respectively. The enhanced activity might be attributed to an increase in the activation entropy by displacing more water molecules by the transition state. The results of molecular dynamics simulations (MD) revealed that the introduction of double disulfide bonds rigidified the global structure of the mutant, which might cause the enhanced thermostability. Finally, the synthesis capacity of the mutant to synthesize phosphatidic acid (PA) was evaluated. The conversion rate of PA reached about 80% after 6 h reaction with wild-type MsPLD but reached 78% after 2 h with mutant S148C-T206C/D225C-A328C, which significantly reduced the time needed for the reaction to reach equilibrium. The present results pave the way for further application of MsPLD in the food and pharmaceutical industries.

## 1. Introduction

In general, specific activity, selectivity and stability are three important evaluation criteria during enzyme mining and engineering for further industrial applications. Due to the specific requirements of processing conditions, we not only expect the enzyme to have high activity at a certain temperature but also to have outstanding stability under the optimal temperature. Therefore, the half-life time (*t_1/2_*) is an important parameter when characterizing the thermostability of an enzyme, which causes much concern. Enzymes with higher *t_1/2_* are most desirable because they display higher kinetic stability and operational stability. Meanwhile, the cost of enzyme preparation is reduced accordingly [1].

In the process of enzyme engineering, directed evolution and rational design are two main strategies that are commonly applied for enhancing the thermostability of specific enzymes. A directed evolution strategy based on error-prone PCR and DNA shuffling has been successfully employed to stabilize various enzymes at elevated temperatures [2]. However, a large number of colonies need to be screened while using these methods, which is both time-consuming and costly [3]. Several computer-assisted rational design approaches are also widely applied to acquire robust biocatalysts, such as B-factor/root mean square fluctuation (RMSF) analysis [3,4], constraint network analysis (CNA) [5], disulfide bond engineering [6], ddg calculation [7], the framework for rapid enzyme stabilization by computational libraries (FRESCO) [8] and combinations of various methods [9]. Among these approaches, disulfide bond engineering is a promising strategy, since disulfide bonds could stabilize enzymes by reducing the entropy of the unfolded forms of proteins and slowing the unfolding rate of the irreversibly denatured process [10,11]. So far, numerous enzymes have been stabilized by using this strategy, such as chitinase [1], alkaline α-amylase [12], amadoriase [13], endoglucanase [14], and D-psicose 3-epimerase [15]. The consistent geometric relationship of native disulfide bonds is revealed based on protein crystal structures [16]. Therefore, if three-dimensional structures of enzymes have been resolved, it would be available to predict residue sites for forming disulfide bonds based on the structural geometric relationship.

Phospholipase D (PLD, EC. 3.1.4.4) belongs to the PLD superfamily. PLD could hydrolyze the distal phosphoester bond of glycerophospholipids, releasing phosphatidic acid (PA) and a free alcohol head group [17]. Physiologically, PA is a crucial intermediate for lipid metabolism and acts as a signaling messenger in metabolic, cellular, and physiological processes in various organisms [18]. In addition, PA receives increasing attention because of its promising prospect of application in the food, cosmetics, and pharmaceutical industries [19]. For example, PA can be employed as an emulsifier and drug carrier due to its structural contained hydrophilic head and hydrophobic tail characters [20]. In addition, PA has been used as a drug to treat various diseases including depression, mental stress, medications, and cancer [21]. PA can also be applied as a dietary supplement to augment muscle strength for athletes and elderly people [22]. Notably, in addition to the hydrolysis reaction, PLD can also catalyze the transphosphorylation reaction to synthesize new or naturally less abundant phospholipids with functional head groups [23]. As a crucial enzyme employed in phospholipid modification, its thermostability has a significant effect on the cost of industrial applications. However, previous studies mainly focused on substrate selectivity, and only a few reports on improving the thermostability of such enzymes based on disulfide bond modification.

In our previous study, a novel phospholipase D from Moritella sp. JT01 (MsPLD) was biochemically characterized, and its crystal structure was resolved (unpublished data). MsPLD showed application potential in the synthesis of PA. However, we found that the half-life of MsPLD was not ideal under its optimal reaction temperature, which indicated that the enzyme was unstable and thus hindered its further applications. In the present study, molecular dynamics (MD) simulation and disulfide bond prediction based on structure were applied to try to improve the stability of this enzyme. During this process, MD simulation was used to determine the flexible regions of MsPLD, and disulfide bonds were introduced to these regions. Two beneficial disulfide bonds were obtained in the first-round screening, then the thermal stability of the enzyme was further improved by the combination of single site mutations. The molecular basis for improving the stability of the enzyme was explained from the molecular perspective with homology modeling and MD simulation. Finally, the efficiency of the mutant based on PA synthesis was evaluated and compared to wild-type MsPLD. The disulfide bridge engineering strategy and corresponding sites for mutation not only provide candidates for improving the thermostability of other PLD enzymes in the superfamily but would pave a way for the further application of MsPLD in the food, cosmetics, and pharmaceutical industries.

## 2. Results and Discussion

### 2.1. Design of Disulfide Bonds Based on MD Simulation Results

The flexible regions of MsPLD were first identified from the MD simulation results. As shown in Figure S1, the N-terminal, C-terminal, region A (T55-P113), region B (L137-V254) and region C (V418-K500) displayed high root mean square fluctuation (RMSF) values, which indicated that these regions were more flexible. Therefore, these corresponding flexible regions were targeted by introducing extra disulfide bonds to acquire a more stable biocatalyst. The potential cysteine mutation sites that might form disulfide bonds in the above corresponding regions were predicted by using online tools DbD2 and BridgeD. Finally, 14 disulfide bonds were designed in these regions (Table 1, Figure S2).

### 2.2. Preparation of Wild-Type MsPLD and Its Mutants

Wild-type MsPLD and its mutants were expressed in *Escherichia coli* (*E.* *coli*) SHuffle T7. As shown in Table 1, only six disulfide bond mutants were successfully expressed, which is probably because the introduction of disulfide bonds could compromise the expressions of proteins [24]. The formation of disulfide bonds is a rate-limiting step of protein folding; these additional disulfide bonds might seriously reduce the rate of protein folding. All of the enzymes were purified with a Ni-NTA affinity column and further desalted with a desalting column. The purity of all samples was evaluated by sodium dodecyl sulfate-polyacrylamide gel electrophoresis (SDS-PAGE) (Figure S3).

### 2.3. Screening of Beneficial Disulfide Bonds

Specific hydrolysis activities of wild-type MsPLD and its mutants were measured by using L-α-phosphatidylcholine (PC) as substrate. As shown in Table 1, the specific activity of wild-type MsPLD was 13.37 U/mg under its optimum temperature of 35 °C. The specific activity of mutant D225C-A328C was 16.41 U/mg, which was 22.7% higher than that of wild-type MsPLD. The specific activities of S63C-V112C and S487C-I550C could not be determined. The specific activities of mutants S148C-T206C were 14.04 U/mg and similar to the wild-type MsPLD (*p* > 0.05), while for the mutant N146C-T206C (11.40 U/mg) and S450C-V552C (11.27 U/mg), the corresponding specific activities were significantly lower than that of wild-type MsPLD (*p* < 0.05).

The *t_1/2_* value is a critical parameter that is commonly used to evaluate the kinetic stability of enzymes. For measuring *t_1/2_* values, each of the mutants was incubated at 35 °C, then cooled on ice for 15 min, followed by measuring the residual activity at 35 °C. As shown in Table 1, the *t_1/2_* value of wild-type MsPLD was 117 min. Remarkably, the *t_1/2_* values of the mutants S148C-T206C and D225C-A328C were 168 and 245 min, respectively, which were 1.4- and 2.1-fold higher than that of wild-type MsPLD.

**Figure 1:**
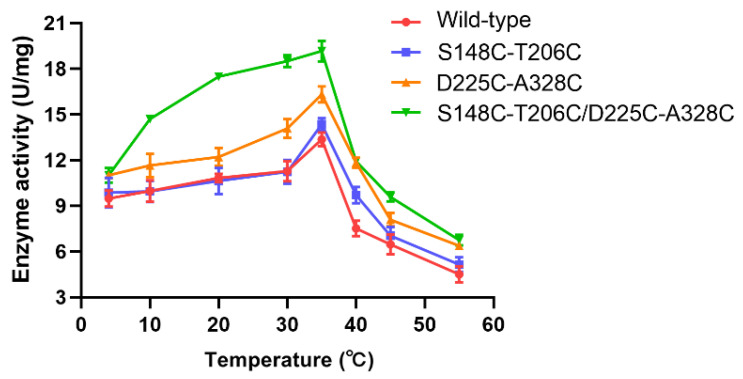
Thermoactivity of wild-type MsPLD and various mutants. Effect of temperature on the hydrolysis activity of wild-type MsPLD and mutants S148C-T206C, D225C-A328C and S148C-T206C/D225C-A328C.

To further investigate the thermodynamic stability of various disulfide bond mutants, the melting temperature (*T_m_*) values were measured by the differential scanning fluorimetry (DSF) method. DSF was determined by monitoring the increased fluorescence intensity of the dye that binds to hydrophobic patches of protein, which were gradually exposed with the unfolding of the protein by heating the temperature [25]. The *T_m_* value was defined as the transition midpoint value between the starting point and the peak point. The *T_m_* value of wild-type MsPLD was 38.3 °C (Table 1). Mutants S148C-T206C and D225C-A328C had *T_m_* values of 43.4 °C and 42.8 °C, respectively, which were 5.1 °C and 4.5 °C higher than that of wild-type MsPLD. These results suggested that mutants S148C-T206C and D225C-A328C had relatively higher thermostability than wild-type MsPLD. The optimal temperatures (*T_opt_*) of mutants S148C-T206C and D225C-A328C were also measured and were consistent with that of the wild type (Figure 1). Moreover, mutants S63C-V112C and S487C-I550C increased the *T_m_* values by 5.5 and 5.4 °C, respectively, although their enzyme activity could not be determined (Table 1). It is interesting to note that the *T_m_* of wild-type MsPLD and mutants S63C-V112C and S487C-I550C (Table 1) were higher than the *T_opt_* (Figure 1), implying that the active-site region of the enzyme unfolds before the total collapse of the overall protein structure. This also means that the active-site region is more flexible than the surrounding structure and that the introduction of disulfide bridges did not delay the unfolding of the active site region as *T_opt_* is not affected.

**Figure 2:**
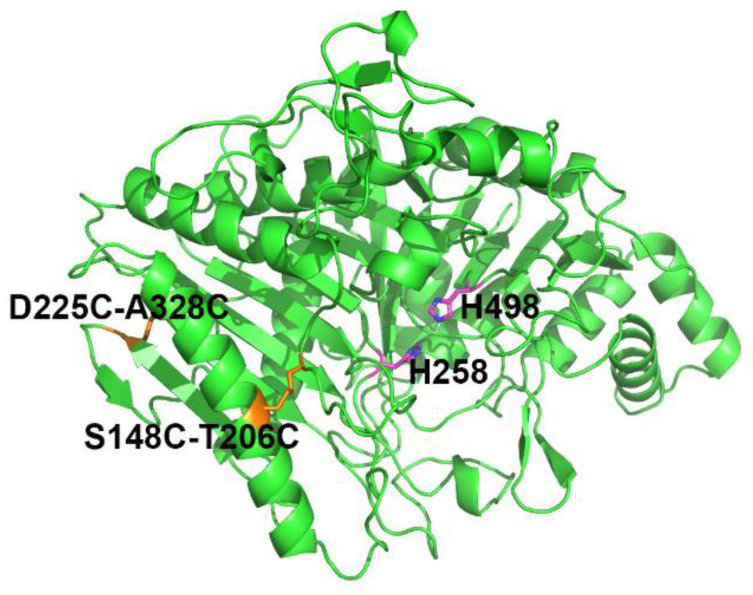
Three-dimensional model of the mutant S148C-T206C/D225C-A328C. The structural model of mutant S148C-T206C/D225C-A328C was built with Modeller, and the crystal structure of wild-type MsPLD (PDB ID 7WU1) was used as the template. Introduced disulfide bonds S148C-T206C and D225C-A328C were shown as orange sticks. The catalytic residues H258 and H498 were shown as magenta sticks.

To verify whether the disulfide bond was formed in the mutants S148C-T206C and D225C-A328C, the free cysteine residues in the enzyme were measured by Ellman’s method [26,27]. The free thiol per molecule of wild-type MsPLD, mutant S148C-T206C and mutant D225C-A328C were 0.012 mol/mol, 0.021 mol/mol and 0.019 mol/mol, respectively (Table S2). The small free thiol per molecule indicated that additional disulfide bonds were successfully introduced into these mutants. The enhanced thermostability of mutants S148C-T206C and D225C-A328C was attributed to the formation of the extra disulfide bonds. Structurally, S148C-T206C connected loop L137-A153 and α-helix E196-V217, while D225C-A328C connected β-sheet L224-V231 and β-sheet T323-N329 (Figure 2).

Disulfide bonds could stabilize the structures of enzymes since it is energetically unfavorable to break the covalent bonds [28]. In addition, the disulfide bond cross-link restricts the motion of regions, which decreases the conformational entropy of the unfolded state [10]. However, while engineering additional disulfide bonds into enzymes, it was not commonly conducive to the enhancement of thermostability [29]. In this study, mutants N146C-T206C and S450C-V552C exhibited decreased thermostability. Similar results were reported on other enzymes, such as haloalkane dehalogenase [30], pullulanase [31], chitosanase [32], and glucose oxidase [33]. It might be attributed to the existing favorable interactions being broken, unfavorable contacts that could be formed by amino acids around disulfide bonds, and the introduction of internal cavities, uncompensated removal of a salt bridge, and exposure of hydrophobic residues at the surface [30]. Therefore, the comprehensive effects of introduced disulfide bonds must be taken into account when attempting to enhance the thermostability of an enzyme.

### 2.4. Combining Beneficial Disulfide Bonds

Beneficial disulfide bonds 148C-T206C and D225C-A328C were further combined into MsPLD to try to obtain a mutant with higher thermostability and activity. Ellman’s test showed that there was no free thiol in the mutant S148C-T206C/D225C-A328C (Table S2), which indicated that these two additional disulfide bonds were both successfully introduced. As shown in Table 1, the *t_1/2_* value of mutant S148C-T206C/D225C-A328C at 35 °C was 369 min, which was 3.1-, 2.2- and 1.5-fold higher than that of wild-type MsPLD, mutant 148C-T206C and mutant D225C-A328C, respectively. Furthermore, the *T_m_* value of mutant S148C-T206C/D225C-A328C was 44.0 °C and was increased by 5.7, 2.2, and 0.6 °C for those of wild-type MsPLD, mutant S148C-T206C, and mutant D225C-A328C, respectively. These results suggested that the beneficial disulfide bonds showed a synergistic effect on the thermostability of MsPLD.

Meanwhile, the temperature dependence of activities was measured for wild-type MsPLD and mutant S148C-T206C/D225C-A328C. As shown in Figure 1, the *T_opt_* value of mutant S148C-T206C/D225C-A328C was 35 °C, which was the same as that of wild-type MsPLD. The *T_m_* value of the wild type was higher than its *T_opt_* value, which indicated the active center might unfold during the early inactivation process [34]. Mutant S148C-T206C/D225C-A328C showed a similar *T_opt_* value to wild type, which suggested the combination of the two disulfide bonds might have less impact on stabilizing the active center. Notably, the enzymatic activity of mutant S148C-T206C/D225C-A328C was 19.18 U/mg at 35 °C, which was increased by 43.5% compared with wild-type MsPLD.

Kinetic parameters of wild-type MsPLD and mutant S148C-T206C/D225C-A328C were further characterized with PC as the substrate under the optimum temperature and pH value (8.0). As shown in Table 2, the catalytic efficiency (*k_cat_/K_m_*) of mutant S148C-T206C/D225C-A328C increased by 36.5%. This suggested that MsPLD displayed a greater catalytic efficiency towards PC substrates while introducing extra double disulfide bonds. In addition, values of activation free-energy (Δ*G^#^*), activation enthalpy (Δ*H^#^*) and activation entropy (Δ*S^#^*) were calculated as described [34]. As shown in Table 2, The Δ*G^#^* of wild-type MsPLD was 58.95 kJ mol^−1^, while that of mutant S148C-T206C/D225C-A328C was 58.40 kJ mol^−1^. Interestingly, there was an increase of Δ*S^#^* on the S148C-T206C/D225C-A328C (−157.49 J mol^−1^ K^−1^) compared to wild-type MsPLD (−175.98 J mol^−1^ K^−1^). Increased Δ*S^#^* could be caused by displacing more water molecules by the transition state, which has been suggested for improving the protein activity [35]. Furthermore, Δ*H^#^* of mutant S148C-T206C/D225C-A328C (9.01 kJ mol^−1^) was higher than that of wild-type MsPLD (4.76 kJ mol^−1^). The increase in Δ*H^#^* of the mutant is structurally accomplished by an increase in the number of enthalpy-related interactions that are broken during transition-state formation. This is likely to generate higher rigidity of the active site of the mutant S148C-T206C/D225C-A328C.

During the natural evolution process of enzymes, it is generally accepted that enzymes display a trade-off between thermostability and catalytic performance while adapting to different environmental temperatures. [36]. It is supported by the fact that cold-adapt enzymes usually show higher catalytic activity and are less stable than those of thermophilic counterparts at low temperatures. In contrast, thermophilic enzymes are highly stable but show less catalytic activity than those of cold-adapt counterparts at high temperatures [37]. However, the thermostability of the enzyme is not always at the cost of its activity [32]. In this study, we synchronously improved the thermostability and catalytic performance of MsPLD, which indicated that it is not always contradictory between improved structural rigidity and enhancing catalytic performance. Similarly, many studies have achieved enhancement in both thermostability and catalytic capacity for molecular modifications of various enzymes, such as peptide amidase [8], ω-transaminases [38], and halohydrin dehalogenase [24]. Our study, through introducing disulfide bonds, might affect the stability of the overall structure instead of the active site, and thus, a trade-off did not occur in the enzyme.

### 2.5. MD Simulation Analysis of Wild-Type MsPLD and Mutant S148C-T206C/D225C-A328C

To explain the enzymatic effect of additional disulfide bonds in mutant S148C-T206C/D225C-A328C, MD simulations were carried out. The structural model of mutant S148C-T206C/D225C-A328C was obtained by using homology modeling with Modeller 9.2 (Figure 2).

**Figure 3:**
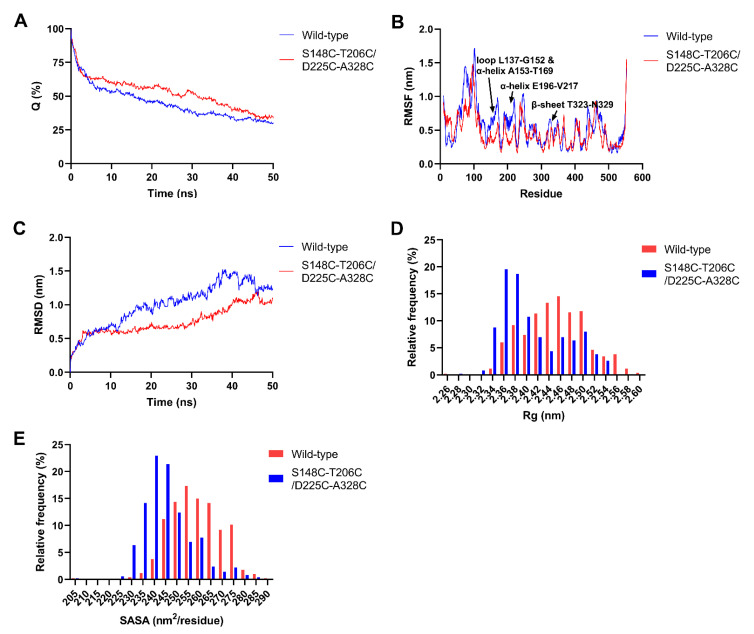
MD simulation analysis of wild-type MsPLD and mutant S148C-T206C/D225C-A328C. (**A**) Time courses of the fraction of native contacts (Q). (**B**) The difference in Cα RMSF of mutant S148C-T206C/D225C-A328C versus that of wild-type MsPLD. (**C**) Time courses of RMSD value. (**D**) The relative frequency distributions of Rg. (**E**) The relative frequency distributions of hydrophobic SASA.

The fraction of native contacts (Q) is a reaction coordinate for measuring the deviation from the native state of structures, while Q closed 0 indicating a state with no resemblance to the native state and unfold more completely [39]. As shown in Figure 3A, the Q of wild-type MsPLD and mutant S148C-T206C/D225C-A328C remained about 30% at 50 ns, which suggested that they unfolded greatly. The RMSF values represent the flexibility of residues under specific condition, high RMSF suggests a higher flexibility. As shown in Figure 3B, residues in loop L137-G152, α-helix A153-T169, α-helix E196-V217 and β-sheet T323-N329 showed relatively lower thermal fluctuations in mutant S148C-T206C/D225C-A328C than in wild-type MsPLD. The root-mean-square deviation (RMSD) value of Cα atoms in wild-type MsPLD rapidly reached 1.5 nm at about 37 ns and then showed a slow decrease (Figure 3C). However, the RMSD of mutant S148C-T206C/D225C-A328C gradually increased and reached 1.1 nm at 50 ns. The results of RMSF and RMSD both indicated that the structure of mutant S148C-T206C/D225C-A328C was more rigid than that of wild-type MsPLD. The radius of gyration (Rg) is a parameter to evaluate the compactness of the protein. Mutant S148C-T206C/D225C-A328C showed a lower Rg value than that of the wild type (Figure 3D). This indicated that mutant S148C-T206C/D225C-A328C displayed less expansion of the structure and kept a more compact structure compared with wild-type MsPLD during the MD simulation process. The hydrophobic solvent accessible surface area (SASA) of wild-type MsPLD was higher than that of mutant S148C-T206C/D225C-A328C (Figure 3E), which suggested that more exposure of the hydrophobic regions occurred in wild-type MsPLD. It is believed that a decrease in the protein–solvent contact area contributes to the protein thermostability [40].

In general, the denaturation of protein happens in two stages. The unfolding of a protein starts with the expansion of its peripheral chains, which is a reversible step, followed by the exposure of hydrophobic core and reactive groups, leading to irreversible inter- and intra-molecular aggregation [41]. Disulfide bonds could increase the free-energy barrier during the enzyme unfolding process, which is conducive to decrease the rate of unfolding [10,11]. In this study, introduced disulfide bonds S148C-T206C and D225C-A328C enhanced the rigidity of MsPLD, which reduced the expansion of peripheral chains and slowed the exposure of hydrophobic regions during the unfolding of the enzyme. Thus, the irreversible inactivation was decreased and the thermostability of MsPLD was enhanced.

### 2.6. Comparison of the PA Production Capacity with Wild-Type MsPLD and Mutant S148C-T206C/D225C-A328C

**Figure 4:**
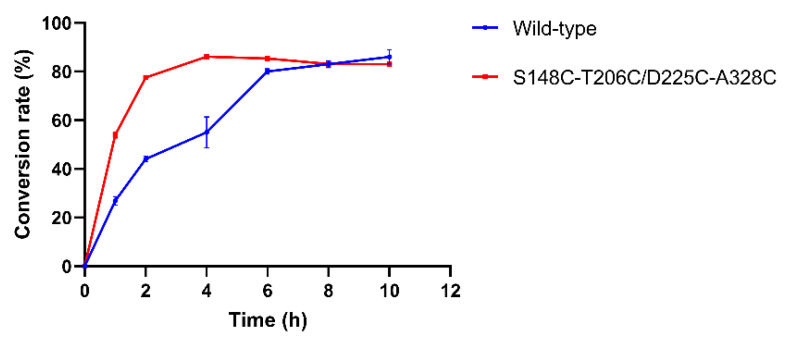
Time course of the production of PA from PC by wild-type MsPLD and mutant S148C-T206C/D225C-A328C.

To demonstrate the practical utility of the mutant in an industrial application, the enzymatic synthesis of PA was carried out. PA was synthesized with wild-type MsPLD and mutant S148C-T206C/D225C-A328C using PC as the substrate. Enzymatic productivity is a measure of product formation or substrate consumption over time under specified reaction conditions. It is the only measure that could reliably summarize the durability and reaction yield of a biotransformation process [42]. As shown in Figure 4, the conversion rates of PA with wild-type MsPLD and mutant S148C-T206C/D225C-A328C both were approximately 86% after 10 h reaction. However, for mutant S148C-T206C/D225C-A328C, the conversion rate of PA reached about 78% at 2 h and then increased slowly. For wild-type MsPLD, the conversion rate of PA reached 80 % after 6 h. It indicated that the mutant enzyme displayed a higher reaction rate. PA conversion rates of both wild-type MsPLD and mutant S148C-T206C/D225C-A328C were about 85% while the reaction reached equilibrium. However, the equilibrium time for the reaction of mutant S148C-T206C/D225C-A328C decreased from 6 h to 2 h compared to the wild-type MsPLD. Productivity is affected by activity, stability, inhibition/activation, and mass transfer. For the previous study, the PA conversion rate was about 90% in a similar reaction by using cabbage PLD while the reaction reached equilibrium, which might result from the enzyme being inhibited by the product of the reaction. A similar product inhibition may have occurred. The present result suggested that mutant S148C-T206C/D225C-A328C might have more potential for the industrial synthesis of PA and bring economic benefits to the industrial process.

## 3. Materials and Methods

### 3.1. Plasmids, Strains, and Materials

Recombinant plasmid pET-21a-MsPLD was constructed by cloning the gene of wild-type MsPLD (GenBank: KXO13223.1) containing an N-terminal His-tag into the vector pET-21a(+) (Novagen, Darmstadt, Germany) with restriction sites *Nde*lI and *Xho*lI. *E. Coli* Top10 and *E. coli* SHuffle T7 were purchased from Weidi Biotech (Shanghai, China). Choline oxidase was prepared as previously described [43]. Horseradish peroxidase and L-α-phosphatidylcholine (PC) were purchased from Sigma-Aldrich (San Diego, CA, USA), Isopropyl β-D-1-thiogalactopyranoside (IPTG) and ampicillin were purchased from Sangon Biotech (Shanghai, China). All other chemicals and reagents were of analytical grade or higher quality.

### 3.2. MD Simulation and Disulfide Bond Design

The MD simulation was used to identify flexible regions of MsPLD. The three-dimensional structure of wild-type MsPLD (PDB ID 7WU1) was used for the MD simulation. Gromacs software package version 2019.6 was utilized for the MD simulation with CHARMM36m force field [44]. The box extended at least 12 Å outside all sides of the surface of the enzyme and was filled with TIP3P water. The negative charge of the system was neutralized with sufficient sodium ions. After energy minimization, the system was gradually balanced by heating the temperature from 0 to 483 K at 1 bar for 1000 ps. Then, the 50 ns MD simulation with a 2 fs time step in the NpT ensemble was carried out. Gromacs tools and MDTraj were used for the trajectory analysis.

The residue pairs for the possible formation of disulfide bonds were predicted with online tools Disulfide by Design 2 (DbD2) [45] and BridgeD [46], which were based on the three-dimensional structure of wild-type MsPLD. For the program DbD2, the χ3 angle and Cα-Cβ-Sγ angle were set as −87° or +97° ± 45° and 114.6° ± 10°, respectively. For the program BridgeD, all parameters were set as default values. Disulfide bonds were designed in flexible regions that were identified with MD simulation.

### 3.3. Site-Directed Mutagenesis of MsPLD

Site-directed mutagenesis was performed by using PrimeSTAR DNA polymerase (TaKaRa, Dalian, China) according to the manufacturer’s instructions. Recombinant plasmid pET-21a-MsPLD was used as the template, and primers used for mutagenesis were listed in Table S1. *Dpn*I was used for destroying the template in the PCR reaction mixture at 37 °C for 1 h. The digestion product was transformed into *E. coli* Top10. Then, positive clones were used for DNA sequence analysis to confirm the mutagenesis.

### 3.4. Expression and Purification of MsPLD and Its Mutants

*E. coli* SHuffle T7 colonies harboring recombinant pET-21a-MsPLD or its mutants were cultivated in LB medium containing 100 μg/mL ampicillin with shaking (200 rpm) at 37 °C until the OD_600_ reached 0.6–0.8. Then, 0.2 mM IPTG was used to induce the protein expression at 16 °C for 20 h. After cultivation, the cells were collected by centrifugation and disrupted by the supersonic treatment. The supernatant was used for the purification with the HisTrap HP column (GE Healthcare, Uppsala, Sweden) according to the manufacturer’s protocol. Buffer A was 50.0 mM Tris-HCl, pH 8.0, containing 500 mM NaCl and 40 mM imidazole and buffer B was 50.0 mM Tris-HCl, pH 8.0, containing 500 mM NaCl and 500 mM imidazole. Subsequently, the sample was desalted into 50 mM Tris-HCl, pH 8.0, containing 500 mM NaCl with a HiPrep 26/10 desalting column (GE Healthcare, Uppsala, Sweden). The purity of the sample was evaluated with 12% SDS-PAGE.

### 3.5. Enzymatic Properties Determination

The hydrolysis activity of wild-type MsPLD and its mutants was measured according to the previous method with minor modifications [47]. The enzymatic reaction mixture consisted of 10.0 µL purified enzyme sample, 50 µL substrate PC (5 mM), and 40.0 µL Tris-HCl buffer (50.0 mM, pH 8.0). The reaction mixture was incubated at 35 °C for 10 min, then the reaction was terminated by heating at 100 °C for 5 min and cooled in ice. Subsequently, 60 μL mixture containing phenol (7 mM), 4-aminoantioyrine (5 mM), choline oxidase (10 U/mL) and horseradish peroxidase (1 U/mL) was added to the enzymatic reaction mixture at 30 °C for 30 min for color reaction, and it was terminated by adding 1% (*w*/*v*) triton X-100. The absorbance of the sample was measured at 490 nm. One unit of enzyme hydrolysis activity was defined as the amount of enzyme required to release 1 μmol choline per minute under the assay conditions.

All samples were diluted in 50 mM Tris-HCl, pH 8.0, containing 500 mM NaCl for determining their *t_1/2_* and melting temperatures. For measuring *t_1/2_*, enzyme samples were incubated at 35 °C with different time intervals, then the residual hydrolysis activity was examined. *T_m_* was measured by the differential scanning fluorimetry (DSF) method as described by Wu et al. [8] In brief, 20 μL of the enzyme (0.3 mg/mL) was mixed with 5 μL 50-fold diluted SYPRO orange dye (Sigma-Aldrich; St. Louis, MO, USA). Then, the change in fluorescence intensity was monitored with a CFX 96 real-time PCR system (Bio-Rad, Hercules, CA, USA) by heating the sample from 25 to 99°C at 1 °C/min. The FRET channel was used. The *T_m_* value was calculated with the Boltzmann equation by using GraphPad Prism 8. The optimal temperature (*T_opt_*) was measured by testing the hydrolysis activity at different temperatures (5, 10, 20, 30, 35, 40, 45, and 50 °C).

The kinetic parameters were measured with various PC concentrations ranging from 1.0 to 3.0 mM at 35 °C and pH 8.0 for 5 min. *K_m_*, *k_cat_* and *k_cat_/K_m_* were calculated by using non-linear regression function of GraphPad Prism 8.0. *E_a_*, Δ*G^#^*, Δ*H^#^* and Δ*S^#^* were calculated as the previous description [35].

### 3.6. Verification of Disulfide Bond Formation

To confirm whether additional disulfide bonds were successfully introduced into MsPLD, Ellman’s test was performed [26]. The amount of free cysteine was measured by assaying quantification of free cysteine by using 5,5′-dithiobis (2-nitrobenzoic acid) (DTNB) according to the previous description [27]. Briefly, 0.5 mL enzyme samples (0.3 mg/mL) dissolved in 50 mM Tris-HCl containing 5 M guanidine were mixed with 2 mL DTNB (0.1 mM) and then incubated at 37 °C for 20 min. The absorbance of samples was measured at 412 nm. The absorbance was converted to the free sulfhydryl group content of the samples with a molar absorbance value of 13,600 M^−1^ cm^−1^. The concentration of the purified enzyme samples was measured with a BCA protein assay kit (Sango Biotech, Shanghai, China) according to the manufacturer’s instructions.

### 3.7. Structure Modeling and MD Analysis of the Mutant

The structural model of the mutant was built through homology modeling with the Modeller 10.2 program, and the crystal structure of wild-type MsPLD (PDB ID 7WU1) was used as the template. The program PyMOL was applied for the visualization of the modeled structure. Then, the MD simulation of the mutant was performed as described above.

### 3.8. Evaluation of the Synthesis Efficiency of PA

PC was used as the substrate to produce PA with wild-type MsPLD and mutants. The reaction system was benzene: aqueous 2:1 (*v*/*v*), 80 mg/mL PC and 150 U enzyme loading. The reaction was performed in a water bath at 35 °C, 800 rpm. Samples were withdrawn every 2 h. Reaction products in the sample were extracted with chloroform: methanol (2:1, *v*/*v*). After the organic phase was evaporated, ethanol was added for precipitation. Then, samples were centrifuged at 10,000× *g* for 10 min to remove the ethanol, and acetone was added to wash the precipitation. After drying, the precipitation was dissolved in the mobile phase and analyzed with high-performance liquid chromatography (HPLC) (Waters, Milford, MA, USA). The HPLC was equipped with an evaporation light detector (Alltech, Deerfield, IL, USA) and a Hyperil GOLD silica column (250 mm × 4.6 mm × 5 μm, Thermo Fisher Scientific, Waltham, MA, USA). Mobile phase A contained methyl alcohol/water/acetic acid (85:15:0.5, *v*/*v*/*v*) with 0.05% triethylamine (TEA), and mobile phase B contained n-hexane/2-propanol/mobile phase A (20:45:20, *v*/*v*/*v*). PA conversion (%) was defined as the percentage of PA produced compared with the initial PC concentration.

## 4. Conclusions

Disulfide bonds were introduced to enhance the thermostability of MsPLD. The best mutant of S148C-T206C/D225C-A328C with two additional disulfide bonds displayed both enhanced thermostability and catalytic performance. Extra disulfide bonds enhanced the rigidity of the overall structure and active site. In addition, during the synthesis of PA, the mutant displayed a higher reaction rate and reduced time for the completion of the reaction, compared to wild-type MsPLD. The engineered MsPLD with improved thermostability and catalytic performance might be a promising biocatalyst for broader industrial applications, especially in the preparation of PA. In addition, the mutation sites might give available information for the molecular modification of other PLDs in the superfamily.

## Figures and Tables

**Table 1:** Summary of the expression, stability, and activity of various MsPLD mutants.

Enzyme	Expression ^1^	Enzyme Activity (U/mg)	*t_1/2_* (min) ^3^	*T_m_* (°C)
MsPLD	+	13.37 ± 0.45	117	38.3
E11C-D505C	-			
F53C-P544C	-			
S63C-V112C	+	n.d. ^2^		43.8
N146C-T206C	+	11.40 ± 0.39	88	/ ^4^
S148C-T206C	+	14.04 ± 0.41	168	43.4
D225C-A328C	+	16.41 ± 0.53 *	245	42.8
G242C-K371C	-			
D249C-G402C	-			
K385C-A421C	-			
A387C-A425C	-			
A423C-V460C	-			
I426C-N480C	-			
S450C-V552C	+	11.27 ± 0.50	95	/
S487C-I550C	+	n.d.		43.7
S148C-T206C/D225C-A328C	+	19.18 ± 0.67 *	369	44.0

^1^ + represents the mutants that were successfully expressed, while -represents the mutants that were not successfully expressed; ^2^ n.d., represents the mutant that shows no enzymatic activity under the standard activity assay; ^3^ The purified enzymes were incubated at 35 °C for designed periods, followed by the standard activity assay; ^4^ /, no signal during thermofluor measurements. For enzyme activity, the statistical analysis was performed with ANOVA, multiple comparisons were performed with LSD, * indicates significant differences at *p* < 0.05.

**Table 2:** Comparison on the kinetic constants of the wild-type MsPLD and mutant S148C-T206C/D225C-A328C.

Enzyme	Enzyme Activity (U/mg)	K_m_ * (mM)	k_cat_ * (s^−1^)	k_cat_/K_m_ * (s^−1^ mM^−1^)	*E_a_* (kJ mol^−1^)	Δ*G* ^#^ (kJ mol^−1^)	Δ*H* ^#^ (kJ mol^−1^)	Δ*S* ^#^ (J mol^−1^ K^−1^)
MsPLD	13.37 ± 0.45	3.44 ± 0.81	10.75 ± 0.09	31.72	7.32	58.95	4.76	−175.98
S148C-T206C/ D225C-A328C	19.18 ± 0.67	2.71 ± 0.89	11.72 ± 0.30	43.29	12.45	58.40	9.01	−157.49

* The kinetic constants were determined at 35 °C.

## Data Availability

Not applicable.

## Supplementary Materials

The supporting information can be downloaded at: https://www.mdpi.com/article/10.3390/ijms231911319/s1.
