# Supplementary material for: Improving Both the Thermostability and Catalytic Efficiency of Phospholipase D from Moritella sp. JT01 through Disulfide Bond Engineering Strategy
Source: Int J Mol Sci. 2022 Sep 26;23(19):11319. doi: 10.3390/ijms231911319 (PMC9570233; doi:10.3390/ijms231911319)
Supplement: Supplementary file 1 [file ijms-23-11319-s001.zip › ijms-1889854-supplementary.pdf]

Supplementary materials

Table S1. Primers used in this study.

Primer	Sequence (5'–3')[a]
F53C-F	TCTGGATACCCACTTCACCTGCGGCACTTCCAG
F53C-R	CTGGAAGTGCCGCAGGTGAAGTGGGTATCCAGA
P544C-F	CTGGAAAAACAGCTGTGGACCTGCATGTGGAAAAACTCCATCCAC
P544C-R	GTGGATGGAGTTTTTCCACATGCAGGTCCACAGCTGTTTTTCCAG
S63C-F	CCAGCGAATTTTACAACAAATGCTTCGACGCGCT
V112C-F	GATGCTAAAAAACTGTGCACCTGTCCGGCTGATAAAATTCTGGA
V112C-R	TCCAGAATTTTATCAGCCGGACAGGTGCACAGTTTTTTAGCATC
S63C-F	CCAGCGAATTTTACAACAAATGCTTCGACGCGCT
S63C-R	AGCGCGTCGAAGCATTTGTTGTAAAATTCGCTGG
N146C-F	GATGGATATCAGCCACCTGTGCCTGAGTTTTTCTAGCGGT
N146C-R	ACCGCTAGAAAAACTCAGGCACAGGTGGCTGATATCCATC
T206C-F	CGCCAGCTGTCTCTGTGCCAGACCAACTACCT
T206C-R	AGGTAGTTGGTCTGGCACAGAGACAGCTGGCG
S148C-F	CAGCCACCTGAACCTGTGTTTTTCTAGCGGTGC
S148C-R	GCACCGCTAGAAAAACACAGGTTCAGGTGGCTG
D225C-F	GCCGGAAGTTAACAACCTGTGTATTACCGTTGGTAGCGTG
D225C-R	CACGCTACCAACGGTAATACACAGGTTGTTAACTTCCGGC
A328C-F	CAACACCTTTGTGACCTACTGTAACGGTCAGTACACCTAC
A328C-R	GTAGGTGTACTGACCGTTACAGTAGGTCACAAAGGTGTTG
G242C-F	CTGATCTCTAACTGCTGCAACAACAACAGCCAGAAAGATGTTCTGC
G242C-R	GCAGAACATCTTTCTGGCTGTTGTTGTTGCAGCAGTTAGAGATCAG
K371C-F	GAACAACGGCGTGCTGGATTGCGATGCGGATCAGAGCGAAG
K371C-R	CTTCGCTCTGATCCGCATCGCAATCCAGCACGCCGTTGTTC
S300C-F	GTCCGATCGCGTGCACCGCCACC
S300C-R	GGTGGCGGTGCACGCGATCGGAC
S342C-F	GGCGCACATCTCCTGCACCTACGTGGC
S342C-R	GCCACGTAGGTGCAGGAGATGTGCGCC
S450C-F	ATCTATAGCTCTGGTTACAACTGCGAATTCGTTTATAACTACC
S450C-R	GGTAGTTATAAACGAATTCGCAGTTGTAACCAGAGCTATAGAT
V552C-F	TGTGGAAAAACTCCATCCACTGTCCGATCAACAACAGCTAAC
V552C-R	GTTAGCTGTTGTTGATCGGACAGTGGATGGAGTTTTTCCACA
I550C-F	CCCGATGTGGAAAAACTCCTGCCACGTTCCGATCAACAAC
I550C-R	GTTGTTGATCGGAACGTGGCAGGAGTTTTTCCACATCGGG
S487C-F	GCACATCAACTTCATCTGCATCAACGGTCGCGAAA
S487C-R	TTTCGCGACCGTTGATGCAGATGAAGTTGATGTGC
E11C-F	CACCACCACCATAGCACCAACTGCCTGGATGTGAACGATATCTAT
E11C-R	ATAGATATCGTTCACATCCAGGCAGTTGGTGCTATGGTGGTGGTG
D505C-F	CCCATAACAAACTGTGGATTGTGTGTGATAAAGTTTTCTACGTTGGCA
D505C-R	TGCCAACGTAGAAAACTTTATCACACACAATCCACAGTTTGTTATGGG
D249C-F	CTGCGGCAACAACAACAGCCAGAAATGTGTTCTGCTGAAC
D249C-R	GTTCAGCAGAACACATTTCTGGCTGTTGTTGTTGCCGCAG
G402C-F	AGCAGGCTCTGTTCTTCAAATGTGCTTTCGGTAAAG
G402C-R	CTTTACCGAAAGCACATTTGAAGAACAGAGCCTGCT
A423C-F	CACCGTTATGGAAGCACTGTGCTCTGCAATCTACAAAGGCG
A423C-R	CGCCTTTGTAGATTGCAGAGCACAGTGCTTCCATAACGGTG
V460C-F	GTTTATAACTACCTGCTGAACTGTCTGCACAAAGCTCCGTACTA
V460C-R	TAGTACGGAGCTTTGTGCAGACAGTTCAGCAGGTAGTTATAAAC
I426C-F	AGCACTGGCGTCTGCATGCTACAAAGGCGTGACC
I426C-R	GGTCACGCCTTTGTAGCATGCAGACGCCAGTGCT
N480C-F	GCTAAAACCTTCCTTGATAAGTGCCTGCACATCAACTTCATCTC
N480C-R	GAGATGAAGTTGATGTGCAGGCACTTATCAAGGAAGGTTTTAGC
A421C-F	ACCATCGATGGCACCGTTATGGAATGCCTGGCGTCTGCAA
A421C-R	TTGCAGACGCCAGGCATTCCATAACGGTGCCATCGATGGT
K385C-F	GAAGTTGCGCGCGTTTATGCGTTCTGCAACGCGACCAAATCTATTAAAATC

K385C-R	GATTTTAATAGATTTGGTCGCGTTGCAGAACGCATAAACGCGCGCAACTTC
A387C-F	CGCGCGTTTATGCGTTCAAAAACTGCACCAAATCTATTAAAATCAGCCA
A387C-R	TGGCTGATTTTAATAGATTTGGTGCAGTTTTTGAACGCATAAACGCGCG
A425C-F	TATGGAAGCACTGGCGTCTTGCATCTACAAAGGCGTGACCG
A425C-R	CGGTCACGCCTTTGTAGATGCAAGACGCCAGTGCTTCCATA

Table S2. Free thiol numbers of the wild-type MsPLD and various mutants.

Enzyme	Free Thiol/ Molecule (mol/mol)
MsPLD	0.012 ± 0.003
S148C-T206C	0.021 ±0.004
D225C-A328C	0.019 ±0.009
S148C-T206C/D225C-A328C	0.015± 0.006

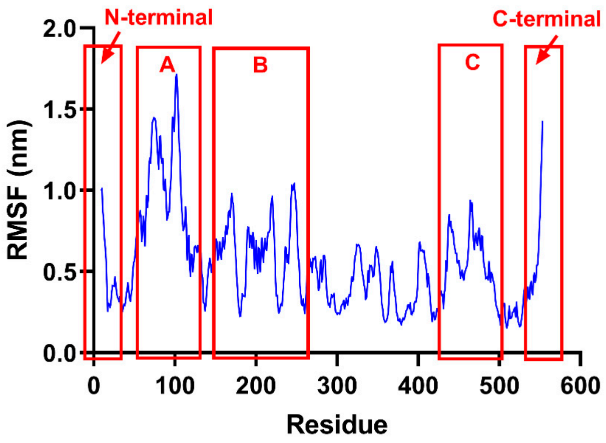

Figure S1. Cα RMSF of MsPLD in the MD simulation at 483 K.

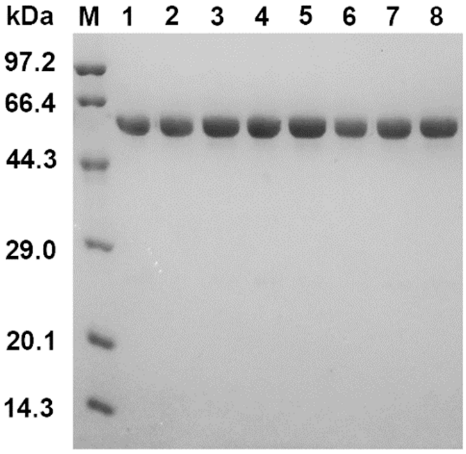

Figure S2. The SDS-PAGE analysis of purified wild-type and mutant enzymes. M is the molecular mass marker. Lane 1 to 8 are Wild-type, S63C-V112C, N146C-T206C, S148C-T206C, D225C-A328C, S450C-V552C, S487C-I550C and S148C-T206C/D225C-A328C, respectively.
